# Complete cytoreductive surgery plus hyperthermic intraperitoneal chemotherapy for gastric cancer with peritoneal metastases: results of a propensity score matching analysis from France

**DOI:** 10.1186/s40880-019-0391-7

**Published:** 2019-08-08

**Authors:** Haibo Qiu

**Affiliations:** Department of Gastric Surgery, State Key Laboratory of Oncology in South China, Collaborative Innovation Center for Cancer Medicine, Sun Yat-Sen University Cancer Center, Guangzhou, 510060 Guangdong P. R. China

Gastric cancer (GC) is the fourth most common malignancy worldwide [1] but almost half of GC-related deaths in the world occur in China [2]. Nearly 20%–30% of GC cases develop peritoneal metastases (PMs) [3]. GC with PMs was once considered as a lethal condition, with death occurring in 53%–60% of advanced GC patients [4]. With advancements in the treatment of gastric cancer, the combination of D2 gastrectomy for advanced GC with comprehensive treatment including complete cytoreductive surgery (CRS) and perioperative chemotherapy, the therapeutic goal has shifted from a palliative to a curative intent [5, 6]. However, only a small number of GC patients with PMs survived longer than 5 years after systemic chemotherapy and complete CRS [7]. Nearly all patients succumb to the PMs within 8 years due to drug resistance [8]. These observations indicate that the effect of systemic treatment on survival improvement is limited, and that systemic chemotherapy alone cannot be curative for them. Adjustments in our approaches to developing new cancer treatments in clinical practice need to occur in order to better prepare ourselves in this new era of precision medicine [9].

Complete CRS plus hyperthermic intraperitoneal chemotherapy (HIPEC) is the only therapeutic strategy that can prolong survival in most peritoneal malignancies, such as malignant peritoneal mesothelioma [10], pseudomyxoma peritonei [11], colorectal cancer [12–14], and ovarian cancer [15]. However, the efficacy of complete CRS-HIPEC remains controversial in GC with PMs. A previous study investigating a cohort of 159 GC patients has reported a median overall survival (OS) of 9.2 months, but only 56% of the cases achieved complete CRS [16]. Within these cases, the observed median OS increased to nearly up to 15 months [16]. These results are undoubtedly directly related to the completeness of CRS. Presently, there is still no evidence regarding the clinical value of complete CRS-HIPEC. In a study recently published in Journal of Clinical Oncology, titled “Cytoreductive surgery with or without hyperthermic intraperitoneal chemotherapy for gastric cancer with peritoneal metastases (CYTO-CHIP study): a propensity score analysis”, Bonnot et al. [17] used propensity score matching analysis with the aim of comparing short- and long-term outcomes between complete CRS alone versus complete CRS with HIPEC in the curative treatment of PMs from GC, conducted by the French National Network for the Treatment of Digestive and Rare Peritoneal Malignancies (BIG-RENAPE) and the French Eso-Gastric Tumors Group (FREGAT).

In this study, the authors analyzed 277 GC cases with PMs who were treated with complete CRS at 19 French centers from 1989 to 2014. Of these cases, 180 underwent CRS-HIPEC and 97 underwent CRS alone. Tumor burden was assessed using the peritoneal cancer index. A Cox proportional hazards regression model with inverse probability of treatment weighting (IPTW) based on propensity score was used to assess the effect of HIPEC and accounted for confounding factors. The two treatment groups were similar after IPTW except for the median peritoneal cancer index (CRS-HIPEC group vs. CRS-group; 6 vs. 2; *P* = 0.003). The median OS of patients from the CRS-HIPEC and CRS group was 18.8 and 12.1 months, respectively. Their corresponding 3- and 5-year OS rates were 26.21% and 10.82%, and 19.87% and 6.43% (CRS-HIPEC group vs. CRS-group, adjusted hazard ratio [HR] = 0.60; 95% confidence interval [CI] = 0.42–0.86; *P* = 0.005), respectively. In addition, the observed 3- and 5-year recurrence-free survival rates were 20.40% and 5.87%, and 17.05% and 3.76% (CRS-HIPEC group vs. CRS-group, HR = 0.56; 95% CI = 0.40–0.79; *P* = 0.001), respectively. However, no significant differences in 90-day mortality were observed between these two treatment groups (CRS-HIPEC group vs. CRS-group, 7.4% vs. 10.1%, *P* = 0.820) or major complication rates (CRS-HIPEC group vs. CRS-group, 53.7% vs. 55.3%, *P* = 0.496). These results indicated that CRS-HIPEC may offer prolonged survival over CRS alone for GC patients with PMs without increasing postoperative morbidity. Furthermore, CRS-HIPEC also provided better outcomes than those previously reported with systemic chemotherapy.

It is difficult to prospectively study the impact of CRS and this may become even more complicated when HIPEC is included because of the rarity of the disease, the major skepticism about the role of surgery in GC with PMs, the need for a strict selection process, and heterogeneity of medical practices. To ensure this study was based on the highest scientific standards, the authors only included patients with a CC-0 or CC-1 CRS (no macroscopic residual or residual nodules ≥ 2.5 mm). Additionally, all these patients were treated at the BIG-RENAPE and FREGAT centers, which are recognized as expert centers for the treatment of peritoneal and gastric malignancies in France. Their findings confirm the crucial role of complete CRS with no postoperative residual because their obtained 5-year OS rate was much higher in patients treated with CC-0 CRS-HIPEC than those with millimeter residuals (24.8% vs. 6.2%, respectively). This suggests that CC-0 CRS should be an absolute requirement before performing HIPEC. Furthermore, the authors also demonstrated that HIPEC helps to kill the residual cancer present, delaying or abrogating intraperitoneal recurrence; as in their study, CRS-HIPEC was statistically associated with major survival benefits in comparison with CRS alone and also showed a trend towards more isolated extraperitoneal recurrences.

The strength of this study is that some measures as follows were taken to reduce potential biases: (1) most of the analyzed data were extracted from prospective controlled databases, which reduced, to a certain extent, some degree of missing data and patients lost to follow-up; (2) the authors made efforts in adherence to strict inclusion criteria regarding PM extent and CRS completeness, such as palliative surgeries were excluded and also excluded patients if their data regarding PM extent or CRS completeness were missing; (3) the use of a propensity score-based on IPTW adjustment which reduces confounding bias and imbalance in covariates and thus potentially offered estimation of treatment effect similar to that of randomized trials; and (4) only variables associated with survival in GC and PMs were selected to obtain the closest to accurate estimation of the effect of HIPEC. Taken together, this study indicates that CRS-HIPEC may represent a valuable therapy option for strictly selected patients and is presently the only treatment that has provided evidence of long-term survival and the possibility of a cure for GC patients with PMs.

## Data Availability

Not applicable.

## Abbreviations

GC: gastric cancer
PMs: peritoneal metastases
CRS: cytoreductive surgery
HIPEC: hyper thermic intraperitoneal chemotherapy
OS: overall survival
BIG-RENAPE: French National Network for the Treatment of Digestive and Rare Peritoneal Malignancies
FREGAT: French Eso-Gastric Tumors Group
IPTW: inverse probability of treatment weighting
HR: hazard ratio
CI: confidence interval
